# South-south collaboration on HIV/AIDS prevention and treatment research: when birds of a feather rarely flock together

**DOI:** 10.1186/s12992-018-0341-1

**Published:** 2018-03-01

**Authors:** Bruna de Paula Fonseca e Fonseca, Priscila Costa Albuquerque, Ed Noyons, Fabio Zicker

**Affiliations:** 1 Center for Technological Development in Health (CDTS), Oswaldo Cruz Foundation (Fiocruz), Av Brasil 4036, 8th floor, room 814, Rio de Janeiro, 21040-361 Brazil; 2 Centre for Science and Technology Studies (CWTS), Leiden University, Leiden, Netherlands

**Keywords:** South-south collaboration, Co-authorship, HIV, Low- and middle-income countries, Scientific collaboration

## Abstract

**Background:**

South-south collaboration on health and development research is a critical mechanism for social and economic progress. It allows sharing and replicating experiences to find a “southern solution” to meet shared health challenges, such as access to adequate HIV/AIDS prevention and treatment. This study aimed to generate evidence on the dynamics of south-south collaboration in HIV/AIDS research, which could ultimately inform stakeholders on the progress and nature of collaboration towards increased research capacities in low- and middle-income countries (LMIC).

**Methods:**

Bibliometric and social network analysis methods were used to assess the 10-year (2006–2015) scientific contribution of LMIC, through the analysis of scientific publications on HIV/AIDS prevention and/or treatment. Five dimensions oriented the study: knowledge production, co-authorship analysis, research themes mapping, research types classification and funding sources.

**Results:**

Publications involving LMIC have substantially increased overtime, despite small expression of south-south collaboration. Research themes mapping revealed that publication focus varied according to collaborating countries’ income categories, from diagnosis, opportunistic infections and laboratory-based research (LMIC single or LMIC-LMIC) to human behavior and healthcare, drug therapy and mother to child transmission (LMIC-HIC). The analysis of research types showed that south-south collaborations frequently targeted social sciences issues. Funding agencies acknowledged in south-south collaboration also showed diverse focus: LMIC-based funders tended to support basic biomedical research whereas international/HIC-based funders seem to cover predominantly social sciences-oriented research.

**Conclusions:**

Although the global environment has fostered an increasing participation of LMIC in collaborative learning models, south-south collaboration on HIV/AIDS prevention and/or treatment research seemed to be lower than expected, stressing the need for strategies to foster these partnerships. The evidence presented in this study can be used to strengthen a knowledge platform to inform future policy, planning and funding decisions, contributing to the development of enhanced collaboration and a priority research agenda for LMICs.

**Electronic supplementary material:**

The online version of this article (10.1186/s12992-018-0341-1) contains supplementary material, which is available to authorized users.

## Background

The growth of scientific research and technological development is crucial for the development of a domestic knowledge base capable of meeting health challenges [1]. International collaboration plays a critical role in this process in low- and middle-income countries (LMIC). Sustainable development of scientific research and technological innovation capacities requires continuous interactions with the world’s science, technology (S&T) and innovation systems [1].

International collaboration on health and development contributes to social and economic progress in LMIC through the provision of universal social basic standards, reduction of extreme inequalities and engagement of LMICs as potential providers of international public goods [2]. Although the traditional official development assistance (ODA) remains a unique and important instrument for development collaboration, the global landscape has changed drastically in the last decades with an increasing participation of LMIC in collaborative learning models [3, 4]. At the same time, many high-income countries (HIC) are turning their attention to LMIC to advance effective solutions for health issues, including the private sector [5].

The World Health Organization (WHO), the Council on Health Research for Development (COHRED) and the Global Forum on Health Research have emphasized that knowledge generated by health research should be utilized to improve health system performance and, ultimately, to promote health and health equity [6–8]. Developing the capacity to effectively carry out health research is essential to strengthen health systems at both national and global levels [9].

LMIC can be vulnerable in some aspects of international scientific collaboration, usually with limited access to the research results and health products arising from these collaborations [10]. Collaboration in health research among LMIC allows sharing and replicating each other’s experiences in finding a “southern solution”, designed to meet their specific needs and complement global solutions, contrasting with the traditional collaboration with HIC [11]. South-South collaboration can facilitate access to strategic knowledge and specific technical skills [12]. More advanced LMIC have addressed common health problems through collaboration between biotech companies and research networks [13]. Although LMIC research collaboration has been assessed by mapping joint scientific publications [14–16], not much is known about the extent and characteristics of research collaborations in common health challenges.

Access to adequate HIV/AIDS prevention and treatment remains one of the common health challenges among LMIC. The uneven geographical distribution of the disease has been related to social determinants of health and income inequality [17]. According to UNAIDS, access to antiretroviral therapy ranges from as low as 16 to 55% in LMIC [18]. The analysis of the LMIC collaboration on HIV/AIDS research may provide an indication of the scientific capacity and potential contribution to the United Nations sustainable development goal 3, target 3, of ending AIDS epidemics by the year 2030 [19]. This analysis may also provide information that could be used to address the goal 17, target 6, of enhancing south-south cooperation and knowledge sharing [19].

The study aimed to generate evidence on the dynamics of south-south collaboration in health research, through the analysis of scientific publications on HIV/AIDS prevention and/or treatment, which could ultimately inform stakeholders on the progress and nature of collaboration towards increased research capacities in LMIC countries. By studying knowledge production, co-authorship, research themes and types, and funding sources, we intended to examine: i) the overall contribution from LMIC to HIV/AIDS prevention and/or treatment research; ii) the frequency of collaboration between LMIC authors (south-south collaborations); iii) the research focus of LMICs in north-south and south-south collaborations; and iv) the research focus supported by LMIC-based funders in south-south collaborations.

## Methods

**Fig. 1:**
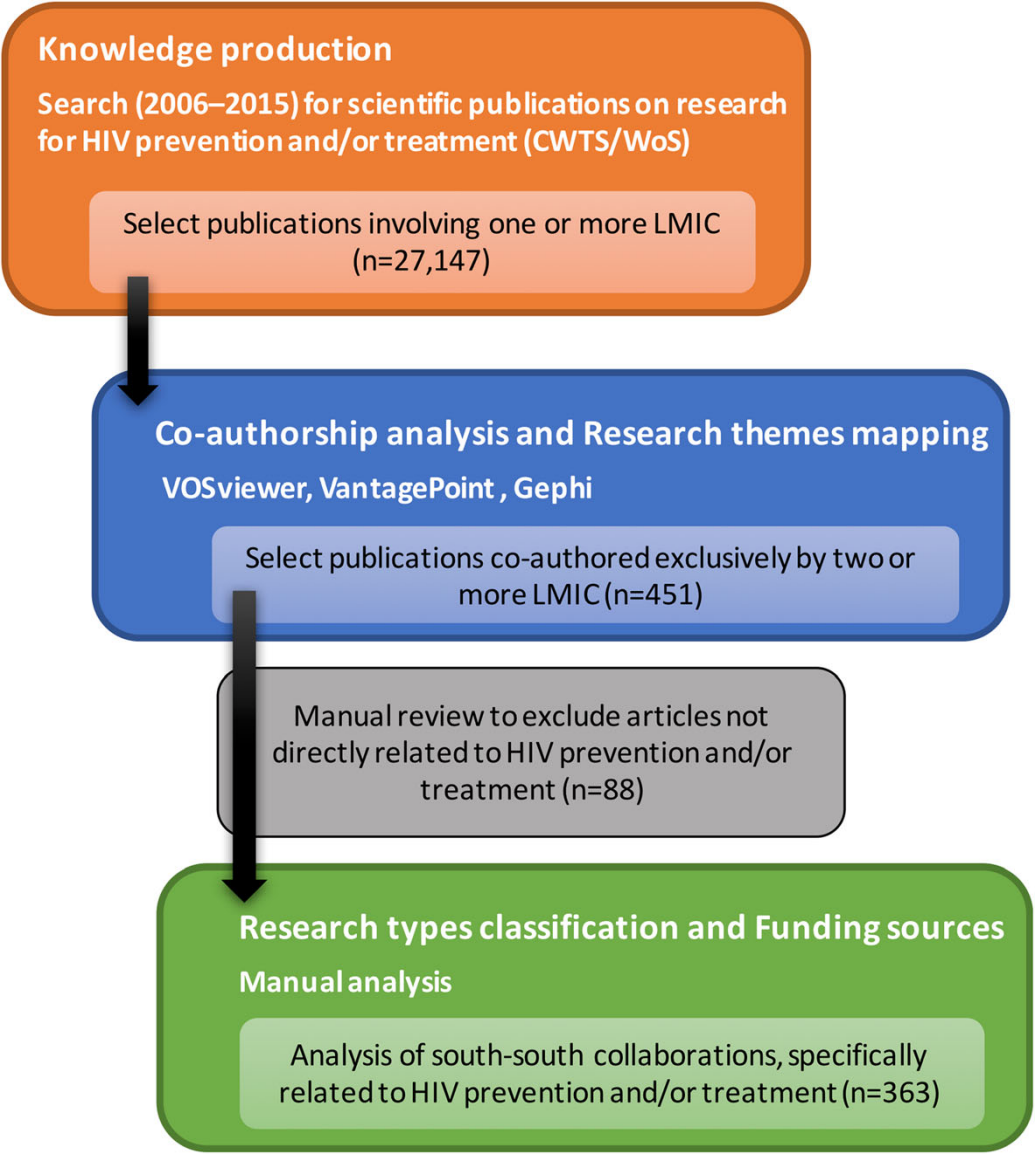
Sampling and selection of papers

Scientific publications were considered herein as evidence of interaction for knowledge production, involving research and training institutions, as well as other components of the scientific and technological infrastructure. Five dimensions oriented the analysis: knowledge production, co-authorship analysis, research themes mapping, research types classification and funding sources. The sampling and selection process is shown in Fig. 1.

### Knowledge production

Information on scientific publications on HIV/AIDS prevention and/or treatment for the period 2006–2015 was retrieved from the bibliometric database of the Centre for Science and Technology Studies (CWTS) of the Leiden University, which is an enhanced structured version of the Web of Science (WoS) database that allowed more efficient data processing.

The 10-year data timeframe was used to be consistent with other bibliometric studies allowing a more accurate review of collaboration efforts. WoS is a well-structured database that has the following advantages: i) covers a large number of academic journals and have high representation of health-related journals; ii) provides information on the affiliations of the authors, allowing the construction of country networks; iii) exports data in text format, compatible with bibliometric analysis software, allowing the systematic cleaning and standardization of data.

Queries were directed to the title, abstract and keywords of the publications. The search query used the terms (“HIV infection” OR HIV OR “hiv infect*” OR “human immunodeficiency virus” OR “human immunedeficiency virus” OR “human immuno-deficiency virus” OR “human immune-deficiency virus” OR “acquired immunodeficiency syndrome” OR “acquired immunedeficiency syndrome” OR “acquired immuno-deficiency syndrome” OR “acquired immune-deficiency syndrome”) AND (prevent* OR vaccin* OR treat* OR cure OR curing OR therap*). The term “AIDS” was not included in the search query as it would retrieve many articles related to development and emergency “aid”, not associated with HIV/AIDS.

In order to evaluate the collaboration pattern of LMIC, data on author affiliation were used as a proxy to retrieve only articles involving at least one author based on an upper- or lower-middle, or low-income country, according to the World Bank definition [20] (*n* = 27,147) (Additional file 1). These represented 31.3% of the total HIV/AIDS prevention and/or treatment research articles in the database.

### Co-authorship analysis

Co-authorship analysis provides a vision of collaboration patterns between individuals, organizations or countries [21]. Co-authorship can represent a formal statement of interaction between two or more researchers. Despite the debate about its meaning and interpretation, co-authorship analysis of scientific publications has been widely used to understand and assess collaboration patterns [16, 22, 23].

Data was imported into the data/text mining software VantagePoint (Search Technology Inc.) to generate co-occurrence matrixes based on the authors’ country of professional affiliation. Affiliations originating from England, Scotland, Northern Ireland and Wales were collectively reviewed as the UK (United Kingdom). These matrixes were imported into the Gephi software [24] to build and visualize co-authorship network graphs.

Publications co-authored by at least one LMIC along with one or more HIC author were considered representations of north-south research collaborations. Publications co-authored exclusively by two or more LMIC researchers were used as a proxy for LMIC south-south research collaboration.

### Research themes mapping

A combined approach of mapping and clustering research topics was used to provide an overview of the research themes contained in the full dataset of retrieved publications. Term maps were constructed using the VOS (visualization of similarities) mapping technique available on the VOSviewer software [25]. The software estimates their “similarity” (affinity) using the “association strength” measure, proposed by Van Eck and Waltman [26], based on the number of co-occurrences of terms in the title or abstract of the same publication. The larger the number of publications in which two terms co-occur, the stronger the terms are considered to be related to each other. Therefore, terms that often co-occur in the same publications are positioned close to each other in a term map while weakly related terms (low co-occurrence) are positioned further away from each other in the map. To identify clusters of related terms, the software uses a weighted and parameterized variant of modularity-based clustering [27]. A theme can be understood as cluster of one or more related terms.

The following HIV-related research themes were identified: i) HIV diagnosis, opportunistic infections and HIV-associated diseases (sensitivity, specificity, T cell count, pneumonia, tuberculosis); ii) Virology and molecular biology (activity, expression, protein, mechanism, interaction); iii) Immunology and vaccinology (vaccine, T cell, antibody, antigen, immune response, vaccination); iv) Drug resistance and virus mutations (mutation, resistance, assay, strain); v) Drug therapy and vertical transmission (efavirenz, nevirapine, concentration, protease inhibitor, breastfeeding); vi) Hepatitis co-infection (HCV, HBV, HBsAg); vii) Human behavior and healthcare (man, sex, service, partner, condom use, stigma); viii) Other sexually transmitted diseases (HPV, cervical cancer, gonorrhea).

### Research types classification

To characterize south-south research collaborations in HIV prevention and/or treatment, all articles co-authored exclusively by two or more LMIC researchers (*n* = 451) were individually reviewed to exclude those articles not directly related to the subject (*n* = 88).

The titles and abstracts of the remaining 363 publications were used to identify the type of research in seven major categories, as shown in Table 1. The first five categories were based on the definitions proposed by the World Health Organization classification of research activities [28]. The last two categories (product development & new technologies, and social sciences research) were created to characterize publications that were not necessarily covered by the previous categories. The categorization was initially done by one of the authors and then cross-checked independently by two other co-authors. Discordant classifications were reviewed together and a final classification agreed by consensus.

**Table 1:** Description of the categories used in the analysis

	Category	Description
1	Health systems research	Examination of healthcare practices, health service delivery (access, barriers and quality) and the structure of healthcare systems;
2	Health policy and strategies	Research on processes used in the implementation of public health initiatives (policies, programs and practices) as well as the contextual factors that affect these processes
3	Clinical research	Clinical and laboratory-based studies conducted in human subjects/samples, including evaluation of treatment regimens intended for human use
4	Basic biomedical research	Laboratory-based research; studies of genes and gene products, molecular, cellular and physiological structures and functions, biological pathways and processes including immune function, bioinformatics, development and characterization of mathematical models
5	Population-based research	Studies of disease surveillance and distribution that track incidence, prevalence, morbidity, co-morbidity and mortality including ongoing monitoring of large scale cohorts, social determinants of health, factors relating to physical environment associated with the cause, risk or development of disease
6	Social sciences research	Research that explores beliefs, attitudes and behavioral perspectives, including interpersonal relations, sexual behavior, risk perceptions, knowledge of the disease
7	Product development & new technologies	Discovery, development and testing of new drugs, vaccines and biopharmaceuticals; testing and evaluation of markers, technologies, devices and kits for diagnosis, prediction, prognosis and monitoring in clinical, community or special settings

### Funding sources

Funding information is important to probe questions related to research funding sponsorship [29]. This information was available in the database from mid-2008 and obtained from the acknowledgments section of the 363 HIV/AIDS prevention and/or treatment publications. The VantagePoint software was used to clean and harmonize funder’s names to minimize ambiguity. Harmonization was done using the highest organization level of a funder.

## Results

### Knowledge production: Contribution of LMIC has steadily increased

**Fig. 2:**
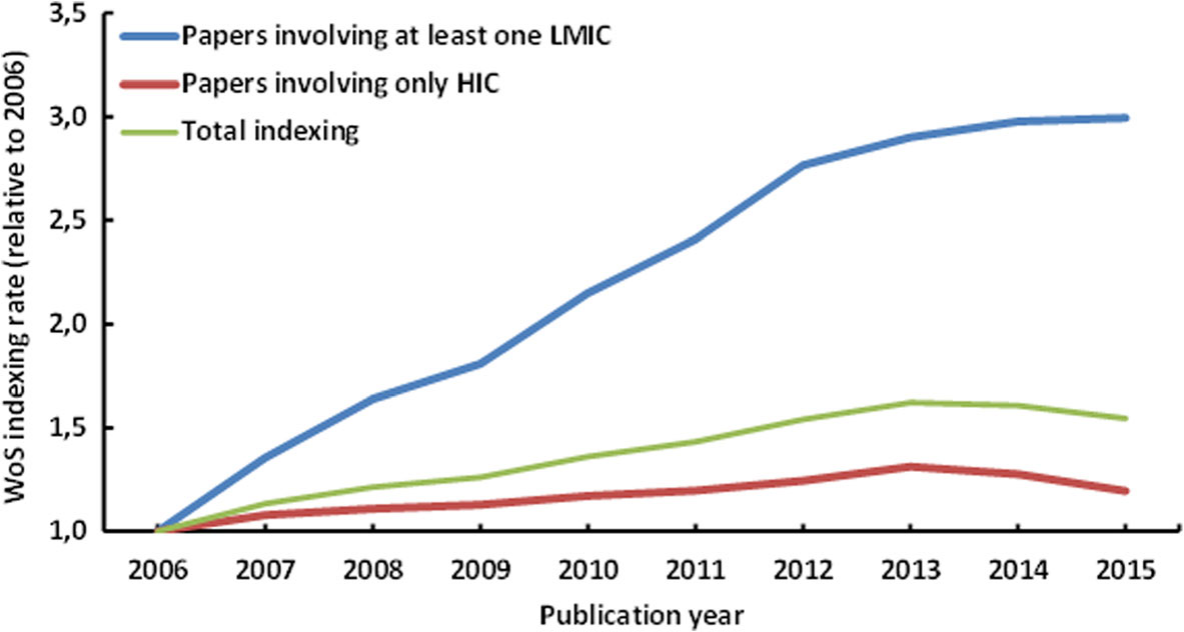
Web of Science indexing rate of scientific publications on HIV/AIDS prevention and/or treatment involving authors from LMIC and HIC (2006–2015). The indexing rate was estimated by the number of indexed papers in each year, relative to the number of HIV/AIDS prevention and/or treatment papers indexed in the database in 2006

The overall contribution from LMIC to HIV/AIDS prevention and/or treatment research, based on the indexing of single-author or collaborative publications, (*n* = 27,147), has steadily increased during the study period (Fig. 2).

Indexing of papers co-authored by at least one LMIC-based scientist have tripled in the past ten years, while articles involving only HIC-based researchers maintained a constant indexing rate. The number of papers of the top ten most active LMIC in HIV/AIDS prevention and/or treatment research is shown in Table 2.

**Table 2:** Top ten most active LMIC in HIV/AIDS prevention and/or treatment research according to first authors’ country of professional affiliation (2006–2015)

Rank	Country	Number of publications
1	South Africa	3714
2	China	2846
3	Brazil	2310
4	India	1869
5	Thailand	825
6	Uganda	674
7	Nigeria	503
8	Kenya	373
9	Mexico	338
10	Argentina	300

### Co-authorship analysis: South-south collaboration was lower than expected

Of the 27,147 publications analyzed, 16,973 (62.5%) were co-authored with other countries; 451 (3%) involving exclusively LMIC researchers and 16,522 (97%) involving collaboration between LMIC authors with at least one HIC.

**Fig. 3:**
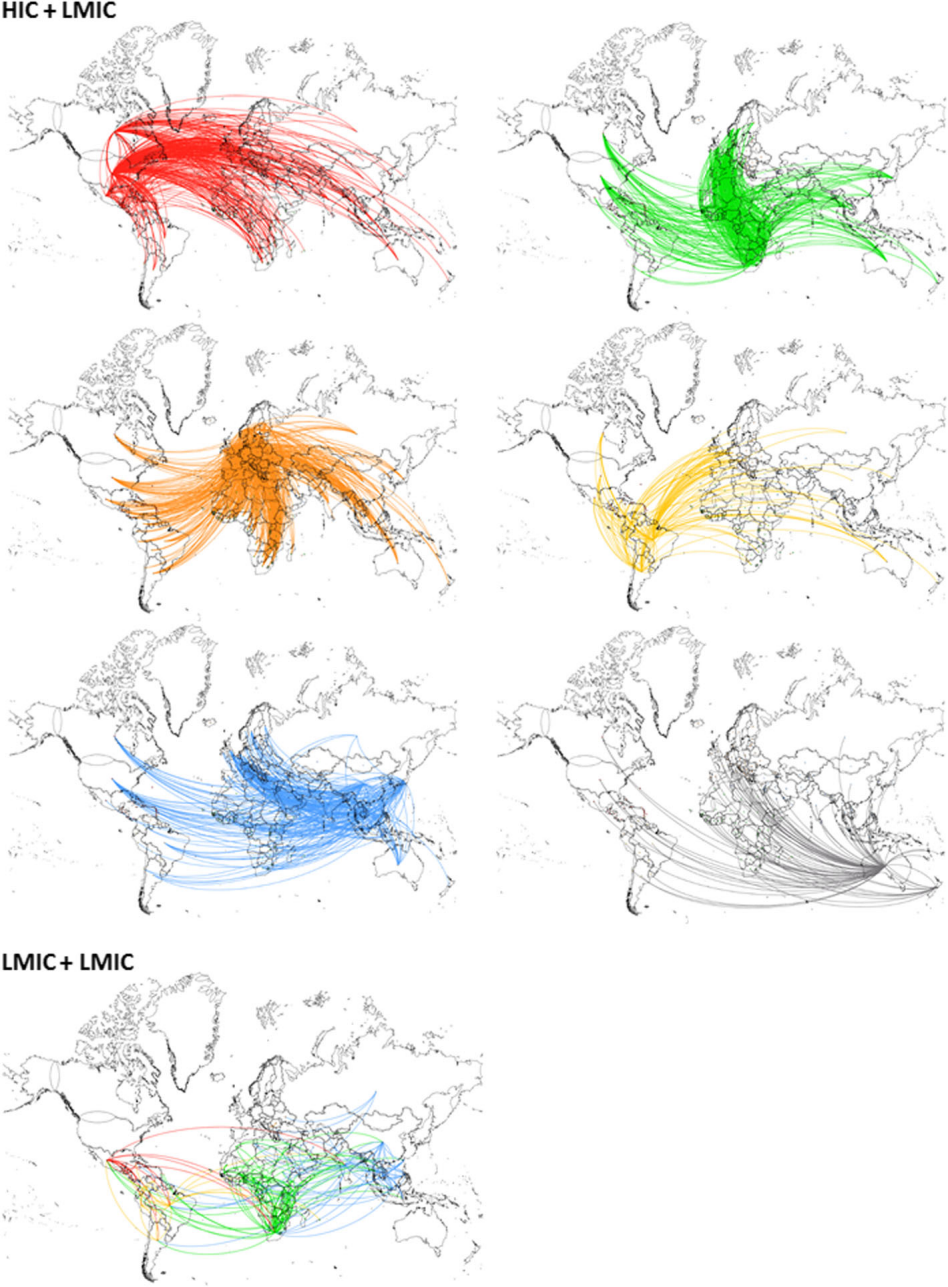
Co-authorship map between HIC and LMIC-based researchers’ in HIV/AIDS prevention and/or treatment research. Country links were mapped based on authors’ affiliations. Each node represents one country and two countries were considered connected if their researchers shared the authorship of a paper. Only relationships between first author and their co-authors are shown. Links are color-coded according to the continent of the first author: North America – red; Africa – green; Europe – orange; South America – yellow; Asia – blue; Oceania – gray

Of the 16,522 papers involving HIC-LMIC collaboration, 14.1% of them (*n* = 2332) involved more than one LMIC. Trilateral collaboration (South-South-North) accounted for 1582 papers, corresponding to 9.6% of all north-south collaboration. Overall display of the co-authorship relations between LMIC and HIC is shown in Fig. 3. The striking difference between the number of HIC-LMIC and LMIC-LMIC links highlighted the small expression of south-south collaboration in HIV/AIDS prevention and/or treatment when compared to north-south.

Looking at the first authors, who presumably made most contributions to a paper, LMIC scientists were the first authors in 37.6% of the publications co-authored with HIC scientists. LMIC first authors in HIC collaborations were mostly from South Africa, accounting for 1751 (10.5%) publications, China for 876 (5.3%) and Uganda for 529 (3.2%). USA-based authors were first authors in most publications in collaboration with LMIC scientists (*n* = 5693 or 34.5%).

Out of 16,522 papers in north-south collaboration, South Africa and USA-based authors were the most frequent collaborators, with 2741 papers (16.5%), followed by South Africa and UK-based authors with 1483 papers (8.9%) and China- and USA-based authors with 1248 papers in co-authorship (7.5%).

Among 451 articles published exclusively by LMIC, South African scientists were the first authors in 25.1% of papers, followed by scientists from India (6.7%), Nigeria (6.0%) and Brazil (5.3%). South Africa, India and Brazil-based authors were the ones that most collaborated with other LMIC researchers. South Africa and Nigeria-based authors had the highest number of collaborative articles among the LMIC scientists (32 papers).

### Research themes mapping: Publication focus varied according to countries’ income categories (LMIC or HIC)

**Fig. 4:**
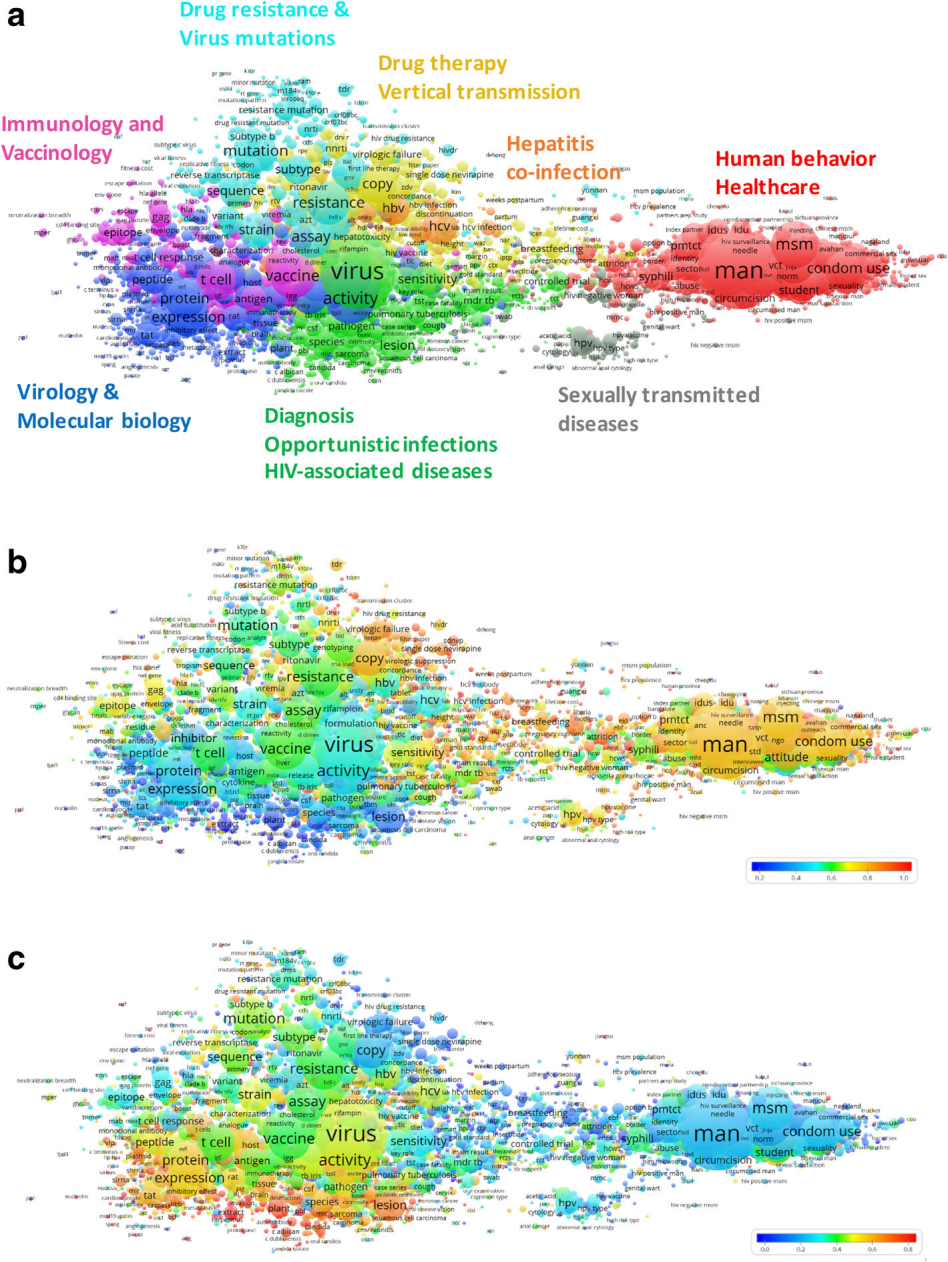
Thematic map of HIV prevention and/or treatment articles authored by low- and middle-income scientists. The map shows 5982 unique terms obtained from titles and abstracts of publications on HIV prevention and/or treatment involving at least one LMIC-based author. The closer two terms are positioned to each other, the stronger their relation. Each term is represented by a circle, where its diameter and label size is proportional to the number of publications that have the corresponding term in their title or abstract. **a** Colors indicate clusters of terms that have co-occurred more frequently in the dataset. **b** and **c** Colors indicate the occurrence of a term in publications co-authored by HIC and LMIC-based scientists (**b**) or in publications involving LMIC authors only (**c**) relative to the whole dataset. Blue represents a low occurrence, green an average occurrence, and red a high occurrence (for the corresponding VOSviewer file see Additional File 2)

The mapping of research themes provided information on the main research topics addressed in the broader field of publications on HIV/AIDS prevention and/or treatment research involving LMIC. The 5982 unique terms identified in titles and abstracts of all 27,137 publications were grouped into eight major clusters (depicted in different colors in Fig. 4a).

The overall thematic mapping was used as a base to overlay publications authored in collaboration with HIC (Fig. 4b) and those exclusively authored by LMIC (Fig. 4c), as single or multi-country collaborations. These maps were color-coded according to the frequency of occurrence of a term relative to the whole dataset. Blue represents a low occurrence, green an average occurrence, and red a high occurrence.

When collaborating among themselves whether between countries or within a country, LMIC scientists’ publications were mainly related to diagnosis, opportunistic infections and other HIV-associated diseases (fungal diseases, tuberculosis etc.) and laboratory-based research, with emphasis on virology and molecular biology. When collaborating with HIC authors, the research emphasis of the publications shifts to human behavior and healthcare, as well as drug therapy and mother to child transmission. Articles with LMIC first authors did not change this pattern significantly.

### Research types classification: South-south collaborations frequently targeted social sciences issues

**Fig. 5:**
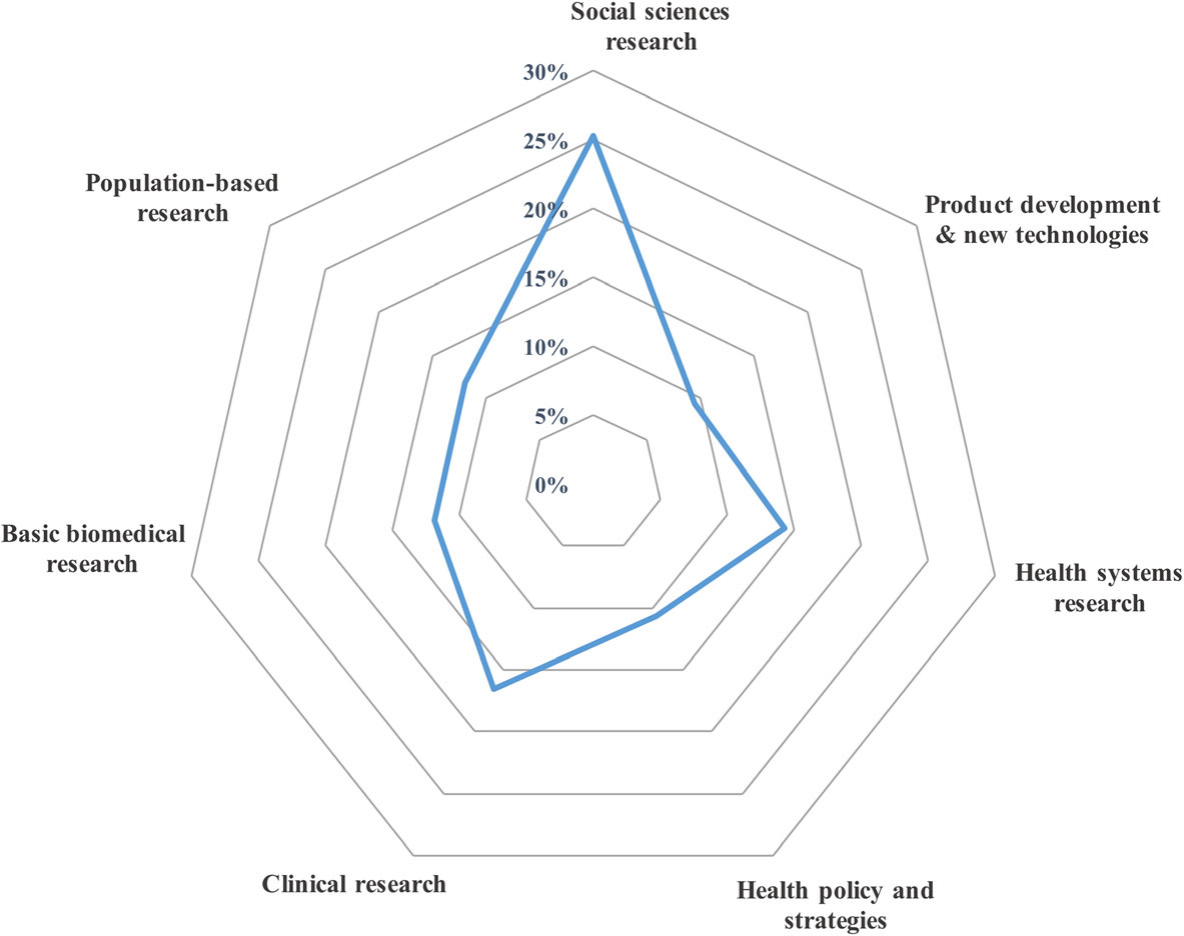
Types of collaborative research (%) published by LMIC-based authors exclusively

Collaboration exclusively between LMIC authors involved 79 countries, but accounted for a small number of articles that were not apparent on the thematic map structure. To characterize south-south research collaborations these 363 publications were classified according to the type of research (Fig. 5).

Social sciences research accounted for 25.3%, clinical research represented 16.5% of publications, followed by health systems research (14.3%), basic biomedical research and population-based research, (11.8% each), and health policy and strategies (10.7%). A smaller proportion (9.4%) of articles were related to product development.

### Funding sources: LMIC-based funders tended to support basic biomedical research

**Fig. 6:**
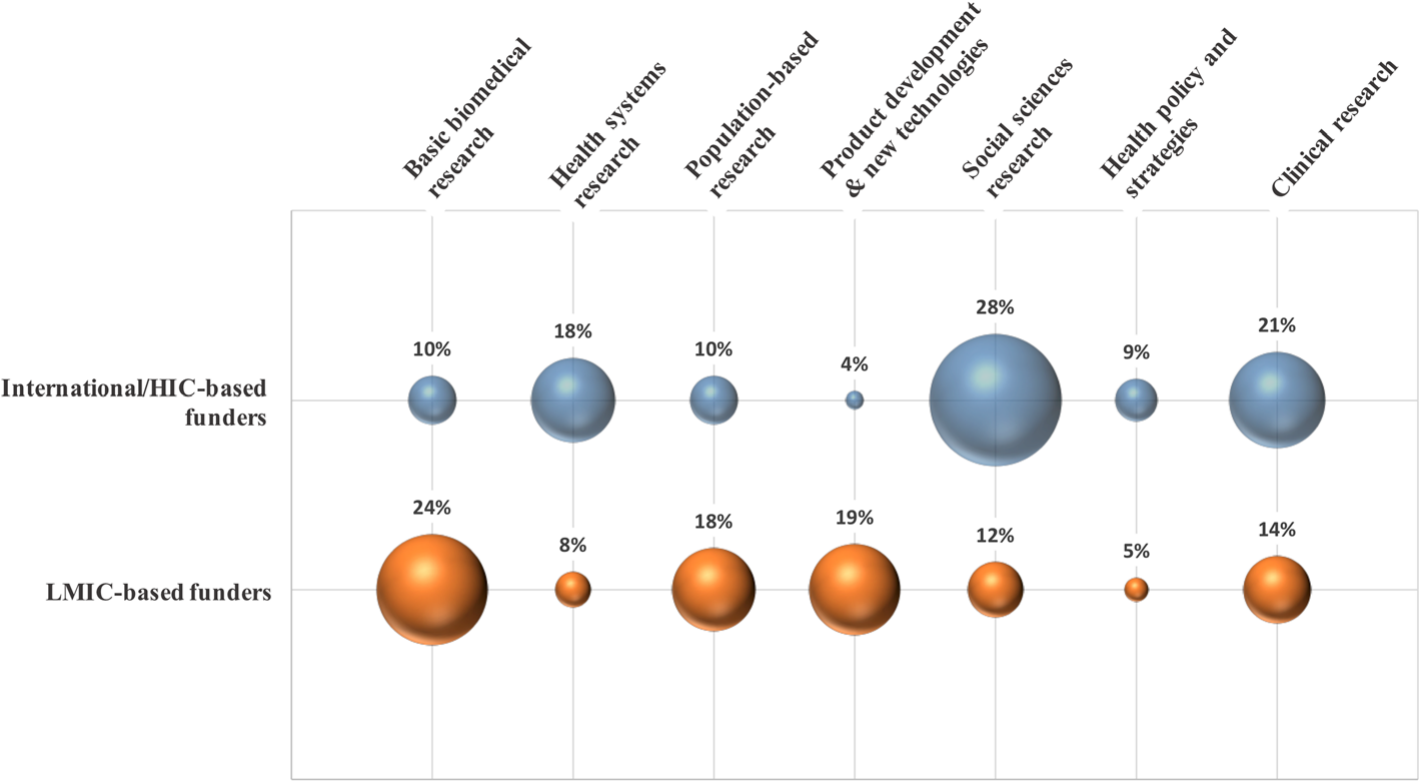
Funders of LMIC collaboration according to the type of research. Bubble sizes are proportional to the number of papers funded of each type

The analysis of funding sources supporting south-south research collaborations revealed that 44% (160 of 363 publications) of publications disclosed at least one funding agency. The Brazilian National Council for Scientific and Technological Development (CNPq) was the most frequent funder acknowledged (*n* = 16), followed by the National Research Foundation of South Africa (NRF) (*n* = 13) and the Medical Research Council of South Africa (*n* = 12). LMIC-based funders were acknowledged in basic biomedical research publications, whereas international/HIC-based funders seem to cover predominantly social science-oriented research, including studies on beliefs, behavior and attitudes related to HIV prevention and/or treatment (Fig. 6).

## Discussion

Over the past two decades, a major development in global health research has been the promotion of collaborative partnerships involving researchers from LMIC. The current paper showed that, over the study period, the contribution of LMIC to HIV/AIDS prevention and treatment research has steadily increased. However, the collaboration south-south collaboration was lower than expected, when compared to north-south. Research themes mapping revealed that publication focus varied according to countries’ income categories. Research types classification showed that south-south collaborations frequently targeted social sciences issues. Additionally, LMIC-based funders tend to support basic biomedical research in south-south collaborations.

Several studies have shown that future savings in healthcare resources can occur if LMIC countries invest in HIV/AIDS prevention [30–32]. The substantial increase in LMIC contributions to HIV/AIDS research is, therefore, a significant finding, bearing in mind that publications on treatment and prevention are interrelated and frequently addressed together. The considerable contribution from South African, Chinese, and Brazilian researchers emphasizes the potential influence of the BRICS on global health [33]. By shaping and leading global health initiatives, these countries have the potential to translate political will into collective action, moving the HIV/AIDS prevention and treatment agenda to better suit their specific needs.

Co-authorship analysis showed that LMIC researchers have collaborated in approximately 62% of all HIV/AIDS prevention and/or treatment papers published, mostly with HIC partners. It has been noted that the cumulative effect of global scientific collaboration has led to the establishment of a core group of collaborating countries that usually excludes LMIC [22], limiting their capacity to efficiently translate and implement scientific knowledge. A study enquiring HIC researchers about the nature of the research contributions made by their LMIC co-authors revealed that their main research inputs were the collection of local-specific data, as well as the contextualization of the findings obtained [15]. Moreover, in most LMIC the process of research strengthening is highly vulnerable to changes in the local economic and political environment, which drives the search for partnerships with more advanced institutions in HIC [1].

In the present study, LMIC-based scientists were the first authors in 37% of papers with HIC collaborators. Regardless of whether this percentage is lower than expected, it has been recognized that researchers from LMICs may have a perception that by allowing HIC authors to take the primary author position (“prestige authorship”), their chances of getting a paper accepted for publication are higher [34]. Lack of guidance with respect to authorship order in a publication and power differentials due to access to research funds could also potentially lead to insufficient recognition of LMIC researchers [34]. However, specifically for HIV research on prevention and treatment, a recent study found a substantial absolute increase in LMIC first and last authorships in cohort studies or randomized trials carried out in LMICs, reflecting a possible expansion of HIV research capacities in these countries [35].

South-South collaboration is often underrepresented in the scientific literature, usually discussed within the context of HIC-LMIC relationships [36]. The high number of single-LMIC papers (*n* = 10,174) suggests that LMIC researchers publish more frequently with other national researchers than with authors from other LMIC. A study on south-south collaboration among health biotechnology firms has shown that more than a quarter (27%) reported collaborations of this type, most of them involving end-stage commercialization activities, rather than research and development (R&D) [13].

The limited collaboration between LMIC scientists has been explained by the insufficient research infrastructure, inadequate human and financial resources [37], weaknesses in manuscript preparation and limited access to scientific literature [38]. Additional reasons that could influence the limited number of LMIC co-authorships are editorial bias against articles by LMIC authors, as previously suggested [39]; linguistic barriers that may affect communication of results [40]; and publication fee models of open access publications [41, 42]. As a strategy to mitigate these effects in LMICs, models offering author mentoring schemes along the lines of AuthorAID [43], combined with differential publication fees and open access platforms, whilst maintaining scientific rigor are likely to play a significant role in addressing these issues. The limited contribution of LMIC research from conception to uptake has been highlighted [44]. The results of the study reinforce the need of international research funders and global health initiatives to better coordinate their efforts to address gaps in research capacity, leadership and partnerships in LMIC [45].

Knowledge maps based on publication data can inform about research trends and the knowledge structure of different research fields [46, 47]. The review of publication themes showed that research focus differed according to the nature of co-authorship. LMIC-HIC collaborations were more focused on human behavior, healthcare, drug therapy and mother to child transmission. With the growing academic field of global health, research on human behavior and healthcare provide HIC scholars with opportunities to engage in field work in high burden LMICs, to expose themselves to new cultures, epidemiological settings, and possibly develop expertise to address existing and emerging challenges in HIV healthcare [48, 49]. This “reverse flow of knowledge and expertise” has an important role in balancing two-way scientific benefits between countries to promote learning processes that can potentially generate effective solutions for global health systems [50].

When collaborating among themselves whether between or within a single country, LMIC papers were more focused on diagnosis, opportunistic infections and laboratory-based research. However, when collaborating with other LMIC researchers, the research focus shifts to the social sciences aspects related to HIV prevention and/or treatment, similar to when collaborating with HIC scientists. LMICs thematic emphasis was coherent with their needs to reduce HIV-associated morbidity and mortality [51, 52]. Social science approaches in HIV prevention and/or treatment are central to assess acceptability, adoption and sustained use of new products/technologies [53]. Engaging with the local social and political contexts of populations and communities at risk is key to understand the needs and processes for shaping health systems.

It was surprising that large proportion of single LMIC papers were targeted to laboratory-based research, since it is usually more expensive and highly dependent on infrastructure and specialized human resources. Indeed, recent studies have shown that LMIC research areas are dominated by biomedical and natural science fields [54, 55]. Limited government and private funding, reduced interest of policy-makers towards R&D, and the “brain drain” make it more difficult for LMIC to contribute to new technological developments. A possible explanation is that domestic S&T activities and training, which are largely publicly funded, are modelled on HIC standards, with little relevance to local needs. We have shown that in south-south collaboration, LMIC-based funders also tend to support basic biomedical research. Priorities of funding agencies may have important influence on research topics addressed by scientists [56].

Collaboration between LMIC-based authors emphasizes socially-oriented research, usually funded by international agencies, indicating a common concern that could be nurtured by local funding agencies as well. As effectiveness of new products/technologies is shown to be dependent not only on the efficacy of the technology, but also on a range of social, cultural and political factors, funding more integrated approaches, combining social sciences with biomedical research could be an important way of tackling the HIV/AIDS pandemic [57]. A more socially-oriented focus would align prevention and treatment strategies with country development objectives, such as education, law reform, gender equality, poverty reduction, community systems, employer practices and health systems/infra-structure [53].

### Limitations

We recognize that co-authorship in publications does not necessarily denote research collaboration, as co-production of knowledge can be different from co-dissemination of knowledge. One must therefore accept a certain level of uncertainty when relying on co-authorship analyses to conclude on scientific collaboration as not all instances of research collaboration will lead to a jointly authored paper, and not all co-authored papers imply that the authors listed have worked together [21]. The WoS used as a source of data covers more than 12,000 scientific journals and has been widely used in the study of LMIC collaboration [16, 54, 58]. However, it is possible that some national, regional or specialized journals were not included in the reviewed database.

## Conclusions

In the past 10 years, there was a substantial increase in LMIC research publications on HIV/AIDS prevention and/or treatment. Nevertheless, within this literature, south-south collaborations were less frequent than expected, emphasizing the need for strategies to foster these partnerships.

LMIC-based funders could be more effective by supporting networks to advance scientific capacity. For HIV/AIDS, and other communicable diseases, research networks can be an efficient way of identifying gaps and synergies, promote collaboration and resource optimization, potentiate results and avoid competition. Public policies and global health initiatives could encourage interaction and networking among LMIC scientists, promoting involvement in international projects and supporting the negotiation of collaborative agreements.

Further studies are required to better contextualize and refine the measurement of research collaboration in LMIC countries. The extent to which international visibility through indexed publications is valued in LMIC to the same degree as the dissemination of local-specific research is yet to be evaluated. Additionally, research on diseases that may not pose challenges to HIC could possibly result in different collaboration patterns.

It is expected that the methods presented here can be used to assess shared intellectual contributions to inform future policy, planning and funding decisions in support to enhanced collaboration and harmonized research agendas in LMICs. The challenge is how LMIC can better align their R&D efforts towards societal needs. The idea is not to favor a single type of partnership, but to understand the different implications on research processes, and subsequently develop and implement the mechanisms through which researchers and society can benefit the most.

## Additional files

Additional file 1: List of articles involving at least one author based on a low- or middle-income country (LMIC). (XLSX 17149 kb)

Additional file 2: VOSviewer map file, depicted in Figure 4. (ZIP 8919 kb)

## Abbreviations

AIDS: Acquired Immunodeficiency Syndrome
HIC: High-income country
HIV: Human immunodeficiency virus
LMIC: Low and middle-income country
WoS: Web of Science
