# Supplementary material for: South-south collaboration on HIV/AIDS prevention and treatment research: when birds of a feather rarely flock together
Source: Global Health. 2018 Mar 1;14:25. doi: 10.1186/s12992-018-0341-1 (PMC5831226; doi:10.1186/s12992-018-0341-1)
Supplement: Supplementary file 2 — VOSviewer map file, depicted in Figure 4. (ZIP 8919 kb) [file 12992_2018_341_MOESM2_ESM.zip › figure/Figure4.pptx]

## Slide 1
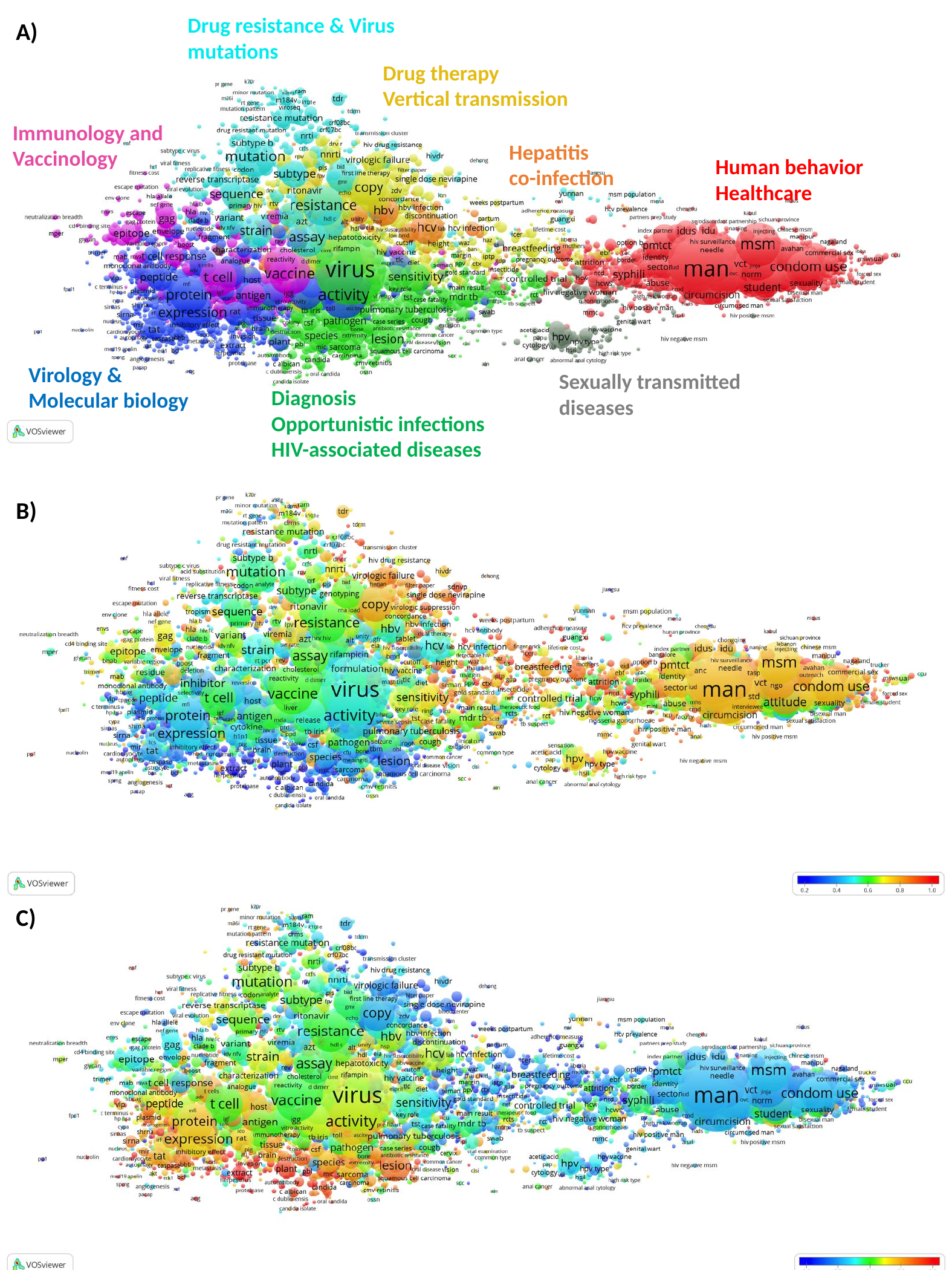

Drug resistance & Virus mutations
A)
Drug therapy
Vertical transmission
Immunology and Vaccinology
Hepatitis
co-infection
Human behavior
Healthcare
Virology & Molecular biology
Sexually transmitted
diseases
Diagnosis
Opportunistic infections
HIV-associated diseases
B)
C)
